# Paper chains: tied visas, migration policies, and legal coercion

**DOI:** 10.1111/jols.12366

**Published:** 2022-05-20

**Authors:** MAAYAN NIEZNA

**Affiliations:** ^1^ Bonavero Institute of Human Rights Faculty of Law Mansfield College University of Oxford Mansfield Road Oxford OX1 3TF England; ^2^ Kent Law School University of Kent Canterbury CT2 7NS England

## Abstract

‘Tied visa’ regimes are labour migration policies that condition migrants’ visas on employment with a particular employer, thus restricting their access to the labour market. This article considers how, under such regimes, control by the state shapes control by employers, and investigates the resemblance between official migration control policies and private means of control and coercion, amounting to forced labour and trafficking. The discussion includes the theoretical analysis and empirical consideration of a case study: the Israeli tied visa regime, regulating migrant workers and Palestinian workers. The consideration of two groups of non‐citizen workers, subject to different but related regimes, enables a novel analysis of the coercive impact of common labour migration policies, and of the justifications offered for such policies. The Israeli Supreme Court demonstrated some commitment to constitutional principles protecting non‐citizens, but later withdrew from these principles and justified tied visas on the grounds that they serve the perceived public interest.

## 1 INTRODUCTION

Adem is a Turkish citizen who was working for a construction company in Israel. After six months of work, he left the company, claiming that he had to work long hours in dangerous conditions, was not paid his full salary, lived in poor conditions, was subject to constant surveillance, and had his passport confiscated. Additionally, the terms of his visa prohibited him from leaving his employer, and thus doing so placed him at risk of deportation. Adem asked to be recognized as a victim of slavery and be granted a new visa that would enable him to change employers. If the means of coercion used against him were his employer's own initiative, he might have been recognized as a victim. However, Adem was subject to a different form of coercion, sanctioned by law; the company was his sponsor, and leaving it meant losing his visa. The Israeli Population and Immigration Authority refused Adem's request, arguing that he had ‘abandoned’ his legal employer and should be deported.^1^

This incident demonstrates the hardships that many migrant workers face. It also demonstrates the shortcomings in how the law responds to the extreme exploitation and coercion of non‐citizen workers. Laws concerning trafficking for labour exploitation and the agencies that enforce them often fail to identify how control and coercion work on a large scale and miss the glaring similarities and connections between the control and coercion exercised by individuals and the control and coercion sanctioned by the state. Threats of detention or deportation, confinement, constant surveillance, restriction of movement, and withholding of documents may directly result from official policies requiring employers to enforce migration control measures. In many situations, means that would be criminalized were they employed as a private initiative are legally mandated for the employers of non‐citizen workers. These disturbing similarities escape not only policy makers but also courts.

The focus of this article is on ‘tied visa’ regimes, coercive migration policies that constitute an important part of temporary labour migration policies in many migrant‐receiving states. Tied visas make migrants’ visas or working permits conditional on employment with a particular employer and prevent workers from freely circulating in the labour market, a key element of what makes wage labour ‘free’ (to the extent that it can be considered ‘free’, given the economic imperative to sell one's labour power).^2^ The less coercive regimes restrict migrants to employment in a specific sector with no access to other sectors of the labour market;^3^ the more coercive tie them to a specific employer. I focus on the more coercive regimes, demonstrating both their proximity to severe violations, such as forced labour and trafficking, and the dilemmas that they pose for courts, which are struggling to protect the rights of non‐citizen workers given the host society's interest in their work. The article considers the moral and legal justifications offered for the use or abolition of tied visa regimes, and the status of non‐citizen workers as reflected in such justifications.

I illustrate and examine the relationship between tied visas, forced labour, and labour exploitation through the specific case study of the Israeli tied visa regime (the ‘Binding Arrangement’), the legal challenges to it, and the changes that resulted from them. I also explore how Israel's Supreme Court first struck down the Binding Arrangement but later withdrew from key principles of its constitutional decision. The analysis of changes, both within and outside the Court, relies on empirical research, drawing on case files, regulations, policy documents, protocols of parliamentary meetings, and qualitative interviews with current and former representatives of state authorities and civil society organizations.

Part 2 introduces the conceptual framework, explains tied visa regimes’ impact on workers’ autonomy, dignity, and rights, and explores possible justifications for such regimes. Part 3 introduces the constitutional challenges to the Binding Arrangement and considers three situations through which we can explore the relationship between different policy areas in practice, and the potential and limits of judicial review: the constitutional case against the entire Binding Arrangement, the case of workers tied to a Turkish construction company in Israel, and the case of migrant caregivers tied to their employers/patients. The two latter cases reflect the weakening of the constitutional case and the role that poor and exploitative conditions play in the Court's assessment of the legitimacy of tied visa regimes. Part 4 looks at the unique political situation in Israel‐Palestine to explore the similarities and differences between the binding of migrant workers and that of Palestinian workers in Israel. Unlike the binding of migrant workers, no court decision has been issued on the binding of Palestinian workers. Significant changes in the binding of Palestinian workers over the last few years and the way in which these came about make it possible to compare the restrictions placed on these two groups of non‐citizen workers, both located in the same territory but subject to related yet distinct regimes. Part 5 concludes.

The Israeli case has unique characteristics, in particular as regards the political situation and its relevance to the regulation of non‐citizen workers. Yet these characteristics, in combination with elements of coercion and exploitation similar to other contexts, facilitate an in‐depth analysis that sheds light on coerced labour in general, and on other tied visa regimes. The discussion of structural coercion and its relationship with private coercion and control, and of the justifications offered for tying workers, is therefore relevant to examples from other contexts – such as Europe, the Middle East, and South‐East Asia – that have been discussed in cases and the literature.^4^

## 2 WORKERS’ RIGHTS, HUMAN RIGHTS, AND JUSTIFICATIONS FOR TIED VISAS

The focus of this article is on tied visa regimes in the secondary labour market, in the ‘dirty, dangerous, demeaning’ jobs where the absence of labour standards, effective enforcement, and collective bargaining leads to poor conditions that local workers reject.^5^ Two related standards, which are recognized in international law and in various domestic legal systems, are particularly relevant to these regimes: freedom from forced labour and the right to free choice of employment. Together, these standards protect workers’ dignity and autonomy and grant some protection from exploitative work.

The international prohibition of forced labour includes ‘all work or service which is exacted from any person under the menace of any penalty and for which the said person has not offered himself voluntarily’.^6^ A ‘menace of penalty’ may arise at the initiation of labour relations, but it may also become part of labour relations later, or restrict workers’ ability to leave employment that they took voluntarily. As Costello notes, a menace of penalty at the exit point is particularly relevant to migrant workers and is not limited to physical violence. Exit from labour relations might be constrained due to the fear of deportation, detention, or loss of status, amounting to menace of penalty.^7^ Deportation, detention, or loss of status reflect legal means of coercion, meaning that legal sanctions either block some choices or impose prohibitive costs on making those choices. The harm that migrants fear and the threats that employers make are related to these legal sanctions. These fears and threats demonstrate the blurred line between legal and illegal, and between documented and undocumented stay. Undocumented migrants fear that without regular status they will be unable to find another employer,^8^ while documented migrants may become undocumented if they leave their employer. Thus, the permits that the workers hold become ‘paper chains’, tying them to their registered employers.

A right closely related to the freedom from forced labour is the right to free choice of employment, an element of the right to work, which is recognized in international human rights law and closely linked to the idea of human dignity.^9^ Free choice of employment reflects two views of human dignity: in a broad sense, as promoting human flourishing, self‐realization, and capabilities,^10^ and in a narrow sense, in the preservation of basic human rights, labour rights, and the guarantee of an adequate standard of living. As explained above and demonstrated in detail below, some tied visa regimes violate human dignity even in the narrow sense. Nonetheless, the broad sense of dignity is also important when it comes to analysing migration policies. Personal development does not cease to be one of the objectives of work when a person crosses a border. Work is imbued with meaning beyond the remuneration that it brings. Inasmuch as free choice of employment is crucial to migrants’ personal and professional development, it is antithetical to their being restricted to filling gaps in specific jobs or sectors. Various aspects of labour relations violate this broad sense of dignity, when workers are treated as a means to create profit without regard for their flourishing and self‐realization. For migrant workers tied to their employers, however, the objectification is worse. This objectification results from temporary migration policies that regulate migrants’ entry, stay, and deportation in order to maintain a supply of labour, which can be (as was said regarding migrant workers in the United States) ‘turned on and off’.^11^ To enable that, aspects of migrants’ humanity unrelated to their labour power are suppressed. Migrants are denied an opportunity to integrate into the host society and the ability to obtain permanent status, denied family unification and family life, and excluded from various social rights.^12^

The prohibition of forced labour deserves particular attention in considering the proximity between tied visas and violations of workers’ rights and human rights. It is important, however, to recognize that forced labour is part of a spectrum of coercion and exploitation ranging from ‘mild’ violations of labour law and the right to work at one end to the extreme situation of trafficking and slavery at the other.^13^ Extreme labour exploitation – a common core of violations at the extreme end of the spectrum (slavery, servitude, forced labour, and trafficking in persons) – is often captured by umbrella terms such as ‘trafficking in persons’ or ‘modern slavery’. I prefer the umbrella term ‘trafficking for labour exploitation’ (or simply ‘trafficking’), and use it to discuss the common core when that is more appropriate than the specific definition of ‘forced labour’. By ‘exploitation’, I refer to the poor and inhumane conditions of employment, as a separate issue from the coercive means used to keep workers in such conditions.

Violation of dignity, violation of autonomy, and exploitation are the elements that constitute forced labour or trafficking for labour exploitation. The combination of these elements is noted in the literature, which highlights the fact that coercion and exploitation are related.^14^ In the case of tied visas, the violation of autonomy is manifold; work is a core human and social activity, and other rights (for example, to just and favourable conditions of work, freedom of association, and even family life) depend on mobility and a certain level of economic independence. The denial of choice of employment restricts autonomy in all of these areas. Even the less restrictive tied visa regimes, which allow workers to change employers within a specific sector, still impose major limitations on migrants’ choice of employment.

The restrictions of the regime itself are accompanied by further violations of autonomy, at the hands of private actors, at various stages of the migration journey. The desire to work abroad and the difficulty in successfully obtaining such work without assistance lead migrants to rely on intermediaries, and to agree to pay high recruitment fees. These fees result in significant debt that migrants are unable to pay without working in the receiving state. In their state of origin, repatriated workers can expect to face poverty, unemployment, or low pay (often the conditions that encouraged migration in the first place), so employment in the state of destination remains their only reasonable option to repay the debt incurred in the course of migrating.^15^ Deportation might result in an unpayable debt, loss of personal or family property used as collateral for loans, or the retribution of criminals involved in the loan scheme.^16^ It is therefore an outcome particularly feared by migrant workers. As the visa is tied to a specific employer, leaving the employer will result in deportation, and deportation will result in debt and risks, adding economic coercion to the legal coercion of the tied visa regime.

In the state of destination, tied visa regimes combine control by the state and control by individual employers, because in return for the benefits that employers gain from tied visas, they are recruited as enforcers of migration control.^17^ To do so, migration laws grant employers control over entry to the state and facilitate their supervision of migrants’ stay and departure,^18^ exacerbating the already substantial imbalance of bargaining power and economic power between workers and employers.^19^ Employers’ control and the coercion resulting from the terms attached to visas are often used to keep workers in poor and inhumane conditions, and employers aware of their workers’ lack of alternatives might exploit this weakness. Workers tied to an employer are denied the minimal safeguard of at least being shielded from the worst employers.^20^ For labour rights, this restriction is particularly problematic, as migrant workers are hard to unionize and therefore do not enjoy the collective power to balance the lack of individual power.^21^ As Albin notes, the allocation of migrants to certain sectors shapes the working arrangements and relations in those sectors, sustaining a sector‐specific inequality.^22^ A focus on sectoral arrangement and regulation is therefore key to understanding the interaction between migration policies, labour market regulation, and employment practices.^23^ In some sectors where retaining workers is a problem, migrants’ tied visas make them desirable to employers and encourage the employers or the sector to depend on them.^24^ The poor conditions in some of the sectors relying on temporary labour migration are the very reason why tied visas are required. The only means to keep workers in some conditions, such as working long hours in hot weather in intense agricultural work, is by leaving them no other choice.^25^ Trapping migrant workers in exploitative jobs that they cannot leave, under inhumane conditions that they cannot improve, violates the core of their dignity and reflects their exploitation.

Can such violations of human rights and workers’ rights be justified? Some common practices that would otherwise meet the definition of forced labour are explicitly excluded from the scope of the prohibition. They include prison labour, military or national service, ‘service exacted in times of emergency’, or ‘normal civil obligations’.^26^ These exceptions can be justified on the grounds of a notion of ‘public good’, which distinguishes between illegitimate private exploitation and legitimate public exaction of labour for limited periods and specific purposes.^27^ Thus, these exceptions reflect the notion that belonging to society entails duties, and that such duties are acceptable as long as they do not impose a disproportionate burden.^28^ Temporary migrant workers are excluded from full membership of the host society, including both the benefits and obligations based on social belonging, so such justifications for their compulsory labour do not apply. Even international labour law permits their binding to an employer for a period of up to two years.^29^ The forced labour of migrant workers in many temporary arrangements, including the one considered below, is justified not because of their membership, but precisely because they are *not* members of the host community. They are invited on a temporary basis, and not allowed to settle in the host state or bring family members with them.

It is the foreignness of temporary migrant workers on which politicians, state agencies, or employers rely to justify restricting their movement. It is argued that restrictions reduce illegal stay and work or address the demand for workers in unattractive jobs. Sectoral tying ensures that sectors characterized by poor conditions do not have to compete with sectors that can offer better conditions. Individual tying protects employers from having to compete with other employers in the same sector, keeping wages low. Tied visas create a more dependable labour force and less vulnerability to unexpected or rapid fluctuations. This justification focuses on employers, but a protectionist justification, protecting local workers from having to compete with migrants, has also been recognized in the scholarship as important.^30^

The exclusion of migrant workers from social membership and its benefits means that they are not the beneficiaries of any public good resulting from the exploitation of their labour. Justifying the violation of migrants’ rights based solely on the interests of the host population is contrary to international standards – and some domestic standards – that protect the rights of non‐citizens and prohibit forced labour.^31^ Some defend certain restrictions of migrant workers’ rights on the grounds of the migrants’ own interests in work, suggesting that employment in the host state subject to some restrictions is better than being altogether denied the opportunity to work abroad and earn more money. Thus, Ruhs and Martin frame the dilemma as ‘numbers vs rights’: a trade‐off between the number of migrant workers given permits to work in the host state, and the rights that these migrants enjoy.^32^ This trade‐off can also justify some restrictions on free choice of employment, though not all forms of tying can be justified in this way. Hence, Ruhs considers the compromise of tying as acceptable only for the short term and rejects prolonged tying as too severe a violation of human rights.^33^ As the next part shows, however, host states sometimes use the language of trade‐offs and migrants’ own interests to justify prolonged tying and severe violations of workers’ rights. Such appeals to the migrants’ own interests are not unique to this case and can be identified in other contexts.

## 3 THE ISRAELI BINDING ARRANGEMENT: DEVELOPMENTS AND JUSTIFICATIONS

Non‐Israeli workers have been a significant part of the Israeli labour market since the late 1960s, when Palestinian workers from the newly occupied territories entered Israeli sectors, primarily agriculture and construction.^34^ Following the first Palestinian uprising in the West Bank and the Gaza Strip (the First Intifada, which began in 1987), there was a shortage of workers. With the growing demands in Israel to rely on migrant workers, temporary labour migration was formally introduced in the early 1990s,^35^ when migrant workers from South‐East Asia and Eastern Europe were recruited.

There were more than 250,000 non‐citizen workers in Israel in 2020, most of them in low‐wage sectors. The largest groups – the focus of this article – are documented migrant workers (about 98,000) and documented Palestinian workers (about 84,000). Most migrant workers work in the care (55,000 workers), agriculture (22,000 workers), and construction (14,000 workers) sectors. Most Palestinian workers (more than 60,000) work in construction, and there are about 5,000 permanent workers and more than 2,500 seasonal workers in agriculture, and smaller numbers in manufacturing, services, health care, and hospitality.^36^

The Binding Arrangement has been a key element in the regulation of labour migration in Israel since its early days in the 1990s. It was officially declared unconstitutional in 2006 but remains important in the employment of non‐citizen workers in some sectors and sub‐sectors. The Israeli case study illustrates three important aspects of the theoretical arguments above. First, it demonstrates the resemblance between forced labour and common labour migration policies. Second, through an assessment of the arguments made for binding in specific cases, it enables a more nuanced analysis of the justifications for tied visa regimes and the weight attributed to them by the judiciary and the executive. Third, it offers a unique opportunity to assess pathways towards the reform of coercive migration and employment policies, and their short‐ and long‐term impact.

### 3.1 The Binding Arrangement: from the early years to the constitutional case

Employing a migrant worker under the Binding Arrangement required a permit conditional on a commitment to only employ the migrant in the specified job and to ensure their departure at the end of employment.^37^ Employers had to ensure the departure of their former worker before they could employ a new worker, and were thus recruited into the state's migration control.^38^ Many withheld the passports of their migrant workers to ensure that their departure was duly recorded so they could be ‘issued’ a new worker.^39^ In one case documented in legal proceedings, employers published a ‘wanted’ advertisement, offering a reward of up to US $3,000 in exchange for ‘information regarding the whereabouts of these workers who escaped from their legal employer’.^40^

The early 2000s saw other means of migration control. The recruitment of new migrant workers into the construction and agriculture sectors was prohibited (due to the ‘Closed Sky Regulation’),^41^ and a campaign for the deportation of migrant workers who had overstayed their visas (including those who had lost their status due to the Binding Arrangement) portrayed them as threatening the state's security and national identity.^42^ Employers therefore had a powerful means of coercion, alongside a strong interest in maintaining control over their workers, as they knew that no new workers would replace those who left.

Following the prohibition of new recruitment, employers holding a permit could request to meet their quota by employing migrant workers detained following their loss of status. Once a successful interview with a detained worker had been concluded, the Foreign Workers Unit would provide employers with the passports and release forms of the workers/detainees and ensure that they were released into the ‘custody’ of the employer.^43^ The level of control and objectification, the selection of workers from detention, and their transfer to a new employer all bear an uncomfortable resemblance to notions of trafficking in persons.^44^ The behaviour of some employers, including on one occasion checking the teeth of detained migrants,^45^ only strengthens that impression. Another procedure from the same period included a calculation of the ‘replacement rate’ for new migrant workers who could replace the tied workers who had ‘abandoned’ their employer.^46^ This commodifying regulation of movement also bears a resemblance to notions of trafficking. Both of these procedures, while no longer in force, demonstrate the conception of labour relations reflected in binding policies, some of which are still in place. The combination of the Binding Arrangement and the restriction of new recruitment under the ‘Closed Sky Regulation’ resulted in a particularly severe form of commodification inherent to tied visas: irreplaceable and valuable workers without any bargaining power. This is a clear difference from fundamental principles of labour relations, where the value of workers often translates into their bargaining power and improved conditions.^47^

The violations of migrant workers’ rights, the means used by employers, and the persistence of coercive and exploitative employment led a coalition of non‐governmental organizations (NGOs) to petition the High Court of Justice (the Supreme Court's title when hearing public law cases) in 2002, in what is known as ‘the *Binding Arrangement* case’.^48^ The petitioners highlighted the structural elements of coercion and exploitation and offered an alternative solution of sectoral binding, which would be better adapted to the conditions in each sector.^49^ While employers’ associations were included as respondents to the petition, the case focused on the government's policy rather than on employers. Interestingly, while the employers’ association in the construction sector adopted the position of the government, the Association of Flower Growers, one of the employers’ organizations in the agriculture sector, supported the position of the petitioners and their call to replace the Binding Arrangement with sectoral binding, citing the interest of employers in direct employment, but also workers’ mobility and bargaining power.^50^

The case was pending before the High Court of Justice for four years. During that time, some changes were made in the different sectors. Of particular importance was the transition to employment by manpower agencies in the construction sector, whereby migrant workers would be registered not with an employer but with an agency that provides their services to contractors. Workers would be able to move between de facto employers, as well as between agencies.^51^ This is still the employment model in the construction sector.

In 2006, the Court delivered its judgment and rejected the Binding Arrangement, with the judges severely criticizing the policy and the position of the state. The judgment was very much rooted in both human rights and labour law arguments. The Court found the Binding Arrangement to violate the basic rights of migrant workers: liberty, autonomy, free will, and freedom of action.^52^ The Court acknowledged:

The essence of the recognition of human dignity as a constitutional right is based on the outlook that the human being – every human being – is an autonomous and free creature, who develops his body and spirit as he wishes, and who writes the story of his life as he chooses.^53^

This point reflects the broad understanding of dignity and free choice of employment discussed above, suggesting that non‐citizen workers have a right to personal development. International standards regarding the right to free choice of employment were cited by the Court.^54^

As for labour law arguments, the Court considered the Binding Arrangement to be contrary to labour protections, and its conclusion was based on ideas from labour law as well as a broad assessment of labour migration. This conclusion took into consideration the economic distress in the countries of origin, the workers’ debt, and the power imbalance between temporary, poor, and unorganized workers and their employers, and between the state and employers.^55^ It described the Binding Arrangement as taking away the economic bargaining power of an already weak party to the employment relationship,^56^ denying workers the opportunity to choose with whom to enter into a contract of employment and negotiate terms of employment and remuneration,^57^ and forcing them into ‘a single choice between a bad alternative and a worse one’.^58^

The language of the judgment reflects an understanding of the notions at the heart of the prohibitions of forced labour and slavery, even if not phrased in the exact terms used in international law. The arrangement was described as ‘compelling a person to work in the service of another against his will’.^59^ The oft‐quoted concurring opinion stated that ‘the restrictive arrangement has created a modern form of slavery’, changing the migrant worker ‘from a subject of the law … into an object of the law, as if he were a kind of chattel’.^60^

International law allows no derogations from or exceptions to the prohibition of slavery, and only rare exceptions to the prohibition of forced labour (such as military or national service, discussed above). Yet in the aftermath of the dramatic judgment, there were many exceptions to the rule prohibiting binding, significant in the many forms that they took, as well as in the absolute numbers of workers still subject to some form of binding. These exceptions allowed – even mandated – the binding of thousands of migrant workers in the construction and care sectors, and (until the end of 2020, as noted below) all of the Palestinian workers in Israel.^61^

In the agricultural sector, migrant workers can change employers formally, but face practical obstacles to doing so. Following a government decision, since 2007 private manpower agencies have coordinated the employment of agricultural workers and are responsible for ensuring that the workers’ rights are protected. This arrangement was meant to facilitate movement between employers and regulate employment.^62^ The government decision also mandated stricter requirements that agencies had to meet, including being a ‘sole purpose’ company and depositing a sum of NIS 500,000 to cover any obligations towards workers or the state.^63^ According to Kurlander, the new regulations were meant to limit the activities of the agencies, reduce their numbers, and involve them in some aspects of workers’ protection.^64^ For the state as regulator, this facilitated inspection and enforcement; it is easier to supervise a small number of agencies (as compared to the larger number of employers), and some functions of regulation and protection are privatized.^65^ The reduction in the number of agencies, however, made movement between employers – which the regulation was supposed to facilitate – harder. Only 11 agencies work in the agricultural sector now. To change employers legally, the cooperation of the agencies is necessary, but agencies are often more committed to the employer than the worker, and do not help with changes, or even intimidate workers to keep them from changing employer. Workers’ isolation, language barriers, and unawareness of their rights inhibit independent job search.^66^

Not only were the principles of the *Binding Arrangement* case poorly implemented, but the judgment was also largely ignored for more than two years after it was issued, and state authorities continued to implement the policies found to be unconstitutional. The petitioners submitted a contempt of court request, and the Court, in two highly critical decisions, accepted their position and instructed the state again to comply with its ruling.^67^ The new decisions summarized the Binding Arrangement in language that yet again reflected aspects of forced labour and trafficking – for example:

Conditioning the presence of the migrant worker in not leaving the employer he came to work for, whose name was stamped on the travel documents, as if the worker was his private property and him [the employer] his owner … in 2002, the Binding Arrangements [sic] were established in their purest form, seeming that only binding in actual chains is more powerful.^68^

The *Binding Arrangement* judgment established general principles and was not limited to particular circumstances, factors, or justifications. However, later developments, and more specific contexts within the case study, led the Court to significantly weaken its position.^69^

### 3.2 Testing the justifications for binding in practice

In the years following the *Binding Arrangement* decision, two cases challenged the Court's previous assessment of the circumstances where binding might be justified: one in the construction sector, and the other in the care sector.

The first case to challenge the *Binding Arrangement* ruling involved construction workers employed in Israel by the Turkish construction company Yilmazlar.^70^ The arrangement between Israel Military Industries Ltd (IMI) and the Turkish Ministry of Defence was signed in 2002. The IMI was to upgrade Turkish Army tanks, and Israel committed to a reciprocal purchase from Turkey. Part of this reciprocal arrangement was for Israel to allow Yilmazlar's employment of 800 Turkish construction workers in Israel. Most of the workers’ wages (minus living costs) were to be sent to Turkey.^71^ The workers would only work for Yilmazlar and could not change employers. The restriction served two purposes. First, it ensured that the arrangement would not open a new route of entry into Israel for migrant workers, who could come to work for Yilmazlar and then change employers. Second, it guaranteed that the number of workers would be maintained so the expected remittances would be sent to Turkey.

In 2004, while the *Binding Arrangement* case was still pending, a petition against the Yilmazlar Arrangement was submitted to the High Court of Justice. It was still pending when the decision regarding the Binding Arrangement was made. The petitioners argued that the principles of the *Binding Arrangement* judgment required the repeal of the ‘binding’ of workers to Yilmazlar. The state argued that the circumstances of the Yilmazlar workers justified a different conclusion. It argued that migrant workers do not enjoy the right to free choice of employment (an argument made before the *Binding Arrangement* judgment); that it had a special interest in the arrangement and the relationship with Turkey; and that the binding was not accompanied by debt, poor conditions, or poor enforcement, so unhappy workers could return to Turkey.^72^ In September 2007, a year and a half after the judgment in the *Binding Arrangement* case, the High Court of Justice accepted the state's position, in a majority opinion. The dissenting opinion was of Judge Levy, who wrote the main judgment in the *Binding Arrangement* case.

The second case concerned workers in the care sector, the largest sector employing migrant workers in Israel. It was argued that this sector has unique characteristics that justify binding. As early as in the first responses to the Binding Arrangement petition, the government justified binding in the care sector on the grounds that persons in need of nursing services might be unable to find a migrant caregiver in a free labour market otherwise, ‘either because of the special difficulty in looking after them relative to other persons in need of nursing care, because of a shortage of funds, or because of the place where they live in Israel’.^73^ This argument rejects workers’ preferences for a less demanding job, higher wages, or a more attractive geographical location as legitimate considerations, and promotes the adoption of regulatory instruments to prevent workers from acting on such considerations. In the *Binding Arrangement* case, the Court did not accept this argument. A few years later, however, in the *Doron* case, care patients and manpower agencies petitioned the Court and demanded an arrangement that would bind caregivers to their sponsors‐patients. The Court was sympathetic to these claims, while the representative of the government correctly argued that the relief sought in the petition meant reversing the Court's ruling in the *Binding Arrangement* case.^74^ As Ben‐Israel notes, the theme of ‘abandonment’ – of workers leaving a dependent patient for an easier job, conflating orderly resignation with unannounced walking out on a dependent patient – was prominent in the arguments of patients and state authorities, as well as in the measures adopted when the patients’ case was pending, and following its conclusion.^75^ These measures included limits on transferring from work in a peripheral area to a more central area,^76^ a regulation against having more than three different employers within a two‐year period,^77^ and the prohibition of switching employers after the caregiver's visa was extended beyond five years and three months, the maximum period for a work visa.^78^ The visas in the latter case could still be extended, but only if the worker remained with the same patient. This latter group was recognized by practitioners whom I interviewed as particularly vulnerable to various forms of abuse, including sexual harassment and trafficking.^79^ Finally, legislation that allowed the Minister of Interior to issue visas for specific subcategories of caregivers was passed but not implemented. It was meant to prevent the option of leaving patients who required more care and harder physical work to undertake easier work.^80^

A comparison of the two judgments – the majority opinion in the *Yilmazlar* case and the position of the Court in the *Doron* case – and between them and the *Binding Arrangement* judgment reflects a reading of the prohibition of forced labour that significantly withdraws from the constitutional principles of the *Binding Arrangement* judgment and its analysis of elements of forced labour. The core of the definition of forced labour, as explained above, is menace of penalty. Legal means of coercion may amount to such menace, especially though not exclusively when accompanied by debt. Poor conditions or exploitation, though not explicit in the definition of forced labour, usually characterize situations of forced labour.^81^

*Lack of coercion* is the first distinction to arise from the *Yilmazlar* case, on the grounds that the agreement between Israel and Turkey prevented high recruitment fees, so the workers did not accumulate debt. In the absence of debt, as argued by the government and accepted by the majority, workers dissatisfied with their working conditions could leave their employer and return to Turkey without fear of the consequences of unpayable debt.^82^ There are a few difficulties with this position. First, as the minority opinion pointed out, the workers had to sign blank promissory notes or bonds, which the company was able to execute. Upon their execution, the company could claim significant sums should the workers leave their work in Israel before the end of their contract and access the workers’ money and property without conditions and for any sum that it saw fit.^83^ The significance of these notes is equivalent to unpayable debt in the country of origin. Second, as noted above, while debt might render deportation more threatening, the threat of deportation alone amounts to menace of penalty, the key element of forced labour. Finally, in the care sector, the recruitment fees paid by workers were not considered by the Court as a reason to reject binding.

A second distinction concerns *lack of exploitation*. Here, too, the Court's analysis is not without difficulties. The majority opinion in *Yilmazlar* accepted that the Turkish and Israeli governments were interested in protecting the workers’ rights, and that both governments employed inspection mechanisms in their respective jurisdictions. It also accepted the government's argument that the conditions of Yilmazlar's workers were better than those of other migrant workers.^84^ The majority was right in distinguishing a convergence of coercion, debt, exploitative conditions, and poor or non‐existent inspection – the circumstances demonstrated in the *Binding Arrangement* case – from a hypothetical case of binding alone. The majority was wrong, however, to dismiss evidence that indicated that Yilmazlar's workers suffered both poor conditions and potential harm following deportation, as analysed in the minority's judgment. The minority recognized that most of the evidence presented by the petitioners, which pointed to low wages, long hours, restrictions of free movement and contact with the outside world, and threats and violence against ‘deserters’, was not refuted by the company.^85^ Restricted movement, threats, and violence are elements of forced labour. More recent testimonies (the company is still operating under the same arrangement) indicate that these elements, as well as other poor conditions typical of forced labour, still characterize the living and working conditions of Yilmazlar's workers.^86^

One could argue that the Court did not ignore these elements but rather preferred the evidence and arguments presented by the state and Yilmazlar regarding the working conditions, even though the judgment itself is not explicit on this point. However, the same argument cannot be made in the *Doron* case. One of the key reasons why the Court justified binding in the specific circumstances of Yilmazlar's workers was because their conditions were argued to be *better* than the general conditions of migrant workers. The argument implied that had that not been the case, binding would be illegitimate. Regarding the care sector, however, the Court accepted the exact opposite position – namely, that binding was justified because the conditions offered were inherently *worse* than other legally available options. A point made by the representative of the NGO Kav LaOved (a workers’ rights organization and the leading petitioner in the *Binding Arrangement* case), that workers leave severely disabled patients because the workload is too much for one person, was dismissed by the judges in a hearing in the *Doron* case.^87^ The Court's assessment also included the argument that in the care sector employers are themselves marginalized and depend on their workers, thus reducing the power gaps between employers and workers, though this argument, too, is not without difficulties.^88^ The seemingly contradictory findings strengthen the point made earlier that coercion and exploitation often go hand in hand. As the minority opinion in *Yilmazlar* pointed out, there is no reason why binding would have been necessary if the conditions offered by the company were indeed so good as compared to the conditions of other migrant workers.^89^

The analysis of the arguments concerning lack of coercion and lack of exploitation suggests that the distinction between the *Binding Arrangement* judgment and the later cases based on these elements is not convincing. There is clearly a contradiction between the Court's respective analyses of coercion and exploitation in the *Yilmazlar* and *Doron* cases, and the *Yilmazlar* conclusion can only stand if some of the evidence presented is rejected. However, the judgment offers no basis for such rejection. How, then, can these judgments be explained, from a Court that so recently emphasized key elements of the prohibition of forced labour and the right to free choice of employment? Part 2 explored not just the prohibition of forced labour, but also the exceptions to this prohibition, and the element of public interest common to these exceptions. Attention to the public interest in the *Yilmazlar* and *Doron* cases complements the analysis of coercion and exploitation.

I suggest that, unlike in the *Binding Arrangement* case, which was concerned with constitutional principles, in the later cases the Court identified the desirable outcome (allowing binding in a specific sector or subsector) and found the reasoning to justify such an outcome. The *public interest* in the binding of certain workers explains what made the outcome desirable. Ensuring affordable care for vulnerable patients is what I referred to earlier as a ‘public good’ – one that migrant workers provide but are not themselves entitled to enjoy. In the *Yilmazlar* case, the Court recognized the state's special interest in a reciprocal arrangement with Turkey, important for international relations and the arms trade (and, implicitly, for national security) – reflecting another public good. Another indication of the perceived public good in this arrangement is the later extension of the ‘Yilmazlar model’ of binding to a foreign company to include more companies. In 2015, six other foreign construction companies (known as ‘execution companies’ – ‘*chevrot bitzua*’ in Hebrew) were sought out to employ their workers in the construction sector in Israel. These posted workers are not allowed to change employers to work for an Israeli company, though the regulation adopted formally allows their mobility between the six companies. This model, too, was approved by the High Court of Justice – notwithstanding the objections and warnings of the manpower agencies employing construction workers, a trade union, and a workers’ rights NGO.^90^

Finally, the cases reflect some consideration of the *workers’ best interests*, though here too some inconsistencies can be identified. The government, the employers, and the Court argued that tied visas serve the workers’ best interests, as otherwise they could not be employed in the host state. In the *Yilmazlar* case, the majority determined that the unique arrangement with the company was the only opportunity for the Turkish workers to be employed in Israel, and that high unemployment in Turkey meant that it was in their interests to do so.^91^ In the *Doron* case, the petitioners referred to migrant caregivers as ‘begging for work’ and to their work visas in Israel, resulting from the efforts of the patients, as ‘realising their dream’.^92^ Such statements echo the trade‐off between numbers and rights; employment in the host state, even if does not fulfil workers’ dreams or offer great conditions, at least presents them with the lesser of two evils. Sectoral binding, which restricts migrant workers’ choice of employment to the least appealing sectors of the labour market, also reflects the trade‐off. The wages are low and the conditions poor, but still better than the opportunities that migrants have at home. Migrants’ broader sense of dignity is violated, but they can improve their lot.

Yet the focus on the choice between two evils implies inevitability and ignores the law's role in restricting choices. For caregivers, freedom to choose another employer in the same sector would have been the lesser evil. The sector is dependent on migrant workers, and sectoral binding would ensure that migrants only work in the sectors approved by the state, but within these sectors they would have some bargaining power to demand the best conditions that the market can bear. It was a policy choice to deny migrants this third and better option and, through legal coercion, force a ‘choice’ between the remaining two evils. This restriction could not be justified on the grounds of either the workers’ best interests or claims of lack of coercion or lack of exploitation. The only convincing justification was the interest of the host state and a particular (vulnerable) group within it. Once this was identified, no further justification was demanded for what a few years earlier had been unanimously determined unconstitutional.

### 3.3 The legacy of the *Binding Arrangement* case

Binding did not disappear once it was declared unconstitutional. The *Binding Arrangement* judgment made no change in reality in the two years that followed it, and only limited change in the long term. Moreover, the declaratory aspect of the judgment was weakened by the Court itself retracting from it in its later decisions in the *Yilmazlar* and *Doron* cases. The change in argumentation and conclusions between the cases reflects the complexity of justifying binding, and migration control in general. On the one hand, the starting point was that binding is illegitimate, except for exceptional cases. Acknowledging the need to provide justification means, at least, recognizing that some standards must be met, even if those standards are not remarkably high. On the other hand, the decisions concerning construction workers and caregivers permitted binding in such significant numbers that it is hard to consider these as mere exceptions to the rule.

The *Binding Arrangement* judgment was not meaningless, but it was not revolutionary either. It was not meaningless for several reasons. First, the Supreme Court's recognition of the rights of migrant workers, their legal personhood, and their dignity was significant, even if policies that do not respect migrants’ rights and dignity are still in place. One of my interviewees, an experienced migrants’ rights lawyer, suggested in this context that the importance of the case was not in changing the specific policy considered, but rather in contributing to a corpus of migration law that recognizes migrants as rights holders, an understanding that could be relevant to the consideration of migrants’ rights in other contexts.^93^ Recognizing migrant workers’ dignity and rights is an important step towards their protection – the first step, not the final one. Kritzman‐Amir, conversely, argues that the recognition of migrants as rights holders was not widely accepted by the legislator, the immigration authorities, or the Court itself – a lack of recognition reflected in later judgments and regulations.^94^

Another important aspect of the *Binding Arrangement* case, suggested by a different interviewee, was the recognition of the link between structural elements and the outcome of forced labour or slavery.^95^ Various arguments made before the *Binding Arrangement* judgment suggested that the state is not responsible for abuse by brokers, agencies, and employers resulting from power gaps or illegitimate practices not directly attributable to the state or its regulations.^96^ Such increased recognition of the responsibility of the state and its policies may have contributed to other important developments in the protection of non‐citizen workers. One such important change was in the employment model of Palestinian workers, discussed below, which required the state to recognize the role of its own policies in rendering non‐citizen workers vulnerable.

## 4 THE BINDING OF PALESTINIAN WORKERS IN ISRAEL

Palestinian workers in Israel, who cross the contested border between an occupied territory and the land of the occupier, experience elements of precarious employment similar to those of migrant workers in Israel and elsewhere: control by employers and authorities, exclusion from social membership, and exploitation of their dependency and vulnerability. Yet their situation is distinct from that of migrant workers, both in the pattern of their movement for labour and in the political context in which they move, which shapes their rights and opportunities.

In terms of movement, most Palestinian workers return to their homes, families, and communities every day, some on the weekends.^97^ They are not ‘migrating’, and are not isolated from support networks. From a migration control perspective, the time between their entry and departure is measured in hours, not years. Even though some work for many years in Israel, they are commuters, not residents. The patterns of their recruitment, movement, and control reflect this important distinction.

In terms of the broader political context, the role of the Israeli state in shaping the choices of Palestinian workers goes deeper than its role concerning migrant workers. Palestinian workers’ availability as a cheaper labour force, their dependency on Israeli employers, the means of control exercised over them, and the unusual aspects of the relationship between their employers and the state authorities are all related to the political and legal reality of the occupation. The imperative to search for work in Israel is strongly linked to Israeli policies restricting the development of the Palestinian economy and labour market, and to Israeli policies that have shaped the dependency of the Palestinian economy on the Israeli economy.^98^ The already weak bargaining power of workers in the secondary market is further weakened by economic dependence, restrictions of free movement, and the political power that Israeli employers have and Palestinian workers lack.^99^ The macro level of the occupation shapes the micro level of regulations and permits.

The situation of Palestinian workers and their binding to specific employers can be compared to that of migrant workers according to the themes considered above: which binding arrangements are adopted in different contexts; how they are justified; and what measures lead to their change, within and beyond legal challenges.

### 4.1 The binding of Palestinian workers: before and after the change of the employment model

Entry permits for Palestinian workers are subject to several considerations, which mostly reflect national security concerns.^100^ For the Israeli regulator, the presence of Palestinian workers means a constant struggle between competing labels: ‘essential workers’ versus ‘dangerous enemies’. Their being ‘bound’ to one employer reflects this tension. The employment of a Palestinian worker in Israel requires two permits: one for the employer, another for the worker.^101^ The permits issued to Palestinian workers and their employers are a means of control, with enforcement privatized and delegated to employers. The employer is responsible for driving Palestinian workers to and from the checkpoint.^102^ The employer is also required to report security concerns,^103^ a requirement inviting abuse by employers to punish workers for demanding their rights.^104^

As permits are subject to a quota, employers who do not use their employment permit may lose it and may be refused if they apply for a new permit in the future. Employers are therefore incentivized to maintain permits that they do not need.^105^ The quota reflects the demand and allocation in the past, which may no longer represent current needs, and some employers need workers but do not hold a permit. At the same time, employers are required to pay Palestinian workers for a minimum number of days per month, reflecting full‐time employment, whether or not they have work for them.^106^ The combination of inefficient allocation and conflicting incentives results in subcontracting the employment of Palestinian workers.^107^ Workers are often illegally employed by someone other than their registered employer. The registered employer charges the subcontractor, who then rolls over the costs to the worker.^108^ The fees are therefore directly related to the tied permits and cannot be isolated from them.

The combination of binding and prevalent illegal employment, especially in the construction sector (where most Palestinian workers in Israel work), has led to many workers having to pay a significant portion of their monthly wages – between 20 and 30 per cent – in illegal brokerage fees to find or maintain a job.^109^ Unlike migrant workers, Palestinian workers do not pay one lump sum to recruiters before travelling to the state of destination. The ‘debt’ pattern of Palestinian workers, like their commuting, is a continuing repetitive experience rather than one major event. The illegal brokerage industry resulted from the employment model of Palestinian workers and the regulation of their stay, but was also one of the factors that eventually led to a change in the employment model, especially in the construction sector.

The problems associated with tied visas have been known and discussed for years in the context of migrant workers. Even though Palestinian workers were also tied to their employers, however, there were no equivalent efforts to address their binding, or even the application of the same terminology and framework to their situation, until a few years later. Unlike the binding of migrant workers, the binding of Palestinians has not been subject to a legal case or judicial review until very recently.^110^ The ground‐breaking decision in the *Binding Arrangement* case did not prompt NGOs to submit a similar petition regarding Palestinian workers, or the government to extend the official abolition of binding to Palestinians. The language of binding that developed in the context of migrant workers slowly spread to Palestinian workers, but with little change in practice.

Nonetheless, attention to the problems resulting from the binding of Palestinian workers, especially illegal brokerage and unregistered employment, was growing, with mounting pressure to address the situation. In 2016, following the recommendation of an inter‐ministerial team, the government decided to allow permit‐holding workers to move between permit‐holding employers at will.^111^ This decision was seen as serving both the interests of the workers and the needs of the market, as well as control measures (reducing the informal transfer of workers without supervision and the undocumented stay of Palestinians in Israel).^112^ Yet it took almost four years for a regulation implementing this decision to be finalized, and it only entered into force in December 2020.^113^ The regulation was published soon after the submission of a petition concerning the binding of Palestinian workers. However, given the short period between the petition and the publication of the new regulations, and the time usually required for drafting such procedures, it is hard to attribute a large role to the petition. A more likely conclusion is that the petition gave the last push to an almost final regulation.

Related to binding is the requirement that the employer constantly monitor the whereabouts of workers, especially of Palestinian workers with an ‘overnight permit’. This permit allows the workers to sleep in Israel between workdays but requires an employer's representative to remain where they sleep throughout the night.^114^ Until mid‐2020, the representative was also required to hold the workers’ documents. Following the COVID‐19 outbreak, regulating the movement of Palestinian workers meant a change from commuters to de facto seasonal workers, subject to the control of the employer for up to two months without going home.^115^ This swift change drew attention to some of the conditions of workers holding an overnight permit, including the requirement to hold documents. Kav LaOved petitioned the High Court of Justice concerning several issues, including the withholding of documents. The petition drew attention to the requirement that employers commit what is otherwise the criminal offence of withholding passports, a practice recognized by the International Labour Organization as indicating forced labour.^116^ In its response to the petition, the state explained that the requirement was part of an old regulation, included in the new COVID‐19 regulations ‘by mistake’.^117^ Unlike the cases concerning migrant workers considered above, no judgment was necessary, and the attention aroused by the petition sufficed for the regulation to be changed. Yet the regulation had been in force for years and did not raise flags for the various officials who encountered it during that time. The fact that such a draconian demand was for years a legal condition for employing Palestinian workers demonstrates the tolerance towards legal means of coercion and coercive policies, even when they clearly resemble elements of forced labour.

### 4.2 Justifications for binding in practice

Since the binding of Palestinian workers includes such coercive practices, both the binding itself and the limited and late proceedings against it call for an explanation. Empirical data suggests several explanations for the lack of legal intervention to abolish the binding of Palestinian workers until recently. One of my interviewees suggested that since the *Binding Arrangement* judgment did not lead to any notable change in practice, there was limited faith in achieving anything through legal proceedings in this context.^118^ The first explicit comparison that I encountered between the binding of migrant workers and that of Palestinian workers, and the demand to abolish the latter like the former, is from July 2008, the same month in which the contempt of court request was submitted,^119^ which may support this explanation. A different kind of explanation can be found in the assessment of justifications for binding, on the grounds of circumstances distinct from those faced by migrant workers.

Some practitioners whom I interviewed suggested that binding was not considered the most urgent problem impacting the lives of Palestinian workers, in practitioners’ assessment of the situation as well as in the complaints of the Palestinian workers themselves.^120^ Furthermore, there was a concern that since the strict control measures applicable to Palestinian workers were justified on the grounds of national security, the government would insist on maintaining binding as a means of control and, if binding were to be rejected, terminate the employment of Palestinians altogether.^121^ These arguments represent the ‘best interests of the workers’ justification considered above. The different reasons for controlling the workers’ movement suggest that, unlike in the case of caregivers considered above, binding may genuinely have been considered a necessary condition to enable Palestinian workers to work in Israel.

This explanation might also be linked to the lack of insurmountable debt and its coercive power. While it was recently suggested that some workers were required to sign promissory notes for six months’ worth of illegal brokerage fees,^122^ earlier documents do not mention any such practice or support a concern of significant debt. A major difference from the situation of migrant workers considered above is that the fees do not accumulate to an unpayable debt. The limited opportunities in the Occupied Territories mean that there is a strong incentive to keep the permit to work in Israel, but this is true for many workers seeking work in stronger economies. The lack of debt was mentioned by some of my interviewees,^123^ as well as informal ways of changing employers, and the knowledge that loss of permit would not result in a ‘one‐way ticket’ to a faraway country was also considered.^124^ All of these factors contribute to a less coercive arrangement.

This point echoes the tension discussed above concerning the workers’ freedom to choose the lesser of two evils. Unlike the binding of migrant workers, the binding of Palestinian workers was justified on the grounds of national security concerns more than labour market concerns, with the intention of controlling the workers’ whereabouts rather than keeping them in uncompetitive jobs. One practitioner whom I interviewed pointed out some of the ways in which the situation of Palestinian workers was better than that of caregivers, emphasizing their relatively high salaries.^125^ Thus, two justifications considered above – perceived lack of exploitation and the best interests of the workers – support the conclusion that addressing binding in this context was less urgent. This conclusion was not phrased this way by my interviewees or in documents to which I had access, but it is my reading of the arguments.

Significantly, both the binding of Palestinian workers and its abolition were justified on similar grounds. Notions of control justified binding the workers to employers, and when the binding was proved to result in illegal transfers and lack of effective control over workers’ presence, the same notions of control, accompanied by concerns for workers’ rights, led to the adoption of another model.

## 5 CONCLUSION

This article has considered tied visa regimes as a form of legal or structural coercion and demonstrated how state control shapes employers’ control and workers’ options and conditions, and how official policies and regulations result in legal means of coercion. As regards legal coercion, it has explored the alarming resemblance between official policies and practices that accompany tied visa regimes and private practices identified with criminal behaviour, amounting to forced labour or trafficking. The comparison between the situation of migrant workers and that of Palestinian workers has demonstrated how different justifications for tied visa regimes can result in different arrangements and compromises. Some of the circumstances of the case study are unique to Israel, but similar arguments have been made in other contexts and regarding other aspects of migration control or restrictions of non‐citizen workers’ rights.

The analysis of legal challenges to the Binding Arrangement has illustrated the potential but also the limits of legal proceedings and judicial review in addressing political questions such as migration policy. It has shown how the decisions of the executive and the Supreme Court stepped back from the leading judgment that struck down the Binding Arrangement as unconstitutional, and how this retreat conformed to neither the principles of the initial constitutional case, nor the international standards on forced labour and free choice of employment. The response to these legal challenges demonstrated, on the one hand, a commitment to constitutional principles and the protection of migrants from coercion, exploitation, and violation of fundamental rights, but on the other hand, the difficulty in maintaining this commitment when a clash with the public good or the interests of vulnerable populations within the host society seems inevitable.

As the case study has demonstrated, in some instances the ‘best interests of the workers’ rationale was used as a thin veil to justify a policy that served the interests of the host society from which the non‐citizen workers are excluded, and led to the disregard of severe coercion and exploitation in practice. Of course, not all arguments concerning the trade‐off between the numbers and rights of migrant workers are so cynical. Here, however, the ‘lesser of two evils’ framing of the choice that non‐citizen workers faced between poverty at home and coercion abroad overlooked the policy choice of the state itself. It was the state's migration policy to deny non‐citizen workers the more appealing option of stronger bargaining power and potential for mobility in the labour market, even when the connection between mobility and entry was based on faulty reasoning. The mobility and stronger bargaining position were denied by the very same migration control policies justified under the pretext of the best interests of migrants.

## Footnotes

1 Immigration Appeal Tribunal (Jerusalem) 1795‐16 *Adem Tavsan* v. *Population and Immigration Authority (PIBA)* (19 July 2017). I represented the petitioner in this case.

2 J. Fudge, ‘(Re)Conceptualising Unfree Labour: Local Labour Control Regimes and Constraints on Workers’ Freedoms’ (2019) 10 *Global Labour J*. 108, at 110.

3 E. Albin, ‘The Sectoral Regulatory Regime: When Work Migration Controls and the Sectorally Differentiated Labour Market Meet’ in *Migrants at Work: Immigration and Vulnerability in Labour Law*, eds C. Costello et al. (2014) 134.

4 Notable examples of tied visa regimes of different durations or restrictions include the artiste regime in Cyprus (*Rantsev* v. *Cyprus and Russia* (2010) Application No. 25965/04 ECtHR (‘the *Rantsev* case’)); the Overseas Domestic Workers visa in the United Kingdom (UK) (V. Mantouvalou, ‘“Am I Free Now?” Overseas Domestic Workers in Slavery’ (2015) 42 *J. of Law and Society* 329; Kalayaan, *Dignity, Not Destitution: The Impact of Differential Rights of Work for Migrant Domestic Workers Referred to the National Referral Mechanism* (2019), at <http://www.kalayaan.org.uk/wp‐content/uploads/2019/10/Kalayaan_report_October2019.pdf>); the Kafala in the Gulf states (A. Khan and H. Harroff‐Tavel, ‘Reforming the Kafala: Challenges and Opportunities in Moving Forward’ (2011) 20 *Asian and Pacific Migration J*. 293, at 295, 297–298; International Labour Organization, *Employer–Migrant Worker Relationships in the Middle East: Exploring Scope for Internal Labour Market Mobility and Fair Migration* (2017) 3, at <https://www.ilo.org/beirut/publications/WCMS_552697/lang–en/index.htm>); and the tied visa regimes in Singapore (S. Yea and S. Chok, ‘Unfreedom Unbound: Developing a Cumulative Approach to Understanding Unfree Labour in Singapore’ (2018) 32 *Work, Employment and Society* 925, at 935); and the Republic of Korea ((23‐2(A) KCCR 623, 2007Hun‐Ma1083, 2009Hun‐Ma230, 352 (consolidated), given 29 September 2011, p. 180, at <http://search.ccourt.go.kr/ths/pr/eng_pr0101_E1.do?seq=1&cname=%EC%98%81%EB%AC%B8%ED%8C%90%EB%A1%80&eventNum=23311&eventNo=2007%ED%97%8C%EB%A7%881083&pubFlag=0&cId=010400>; South Korean NGO Coalition, *Republic of Korea NGO Alternative Report to the UN Committee on the Elimination of Racial Discrimination* (2018) 33, at <https://tbinternet.ohchr.org/Treaties/CERD/Shared%20Documents/KOR/INT_CERD_NGO_KOR_32854_E.pdf>).

5 J. Fudge and J.‐C. Tham, ‘Dishing Up Migrant Workers for the Canadian Food Services Sector: Labor Law and the Demand for Migrant Workers’ (2017) 39 *Comparative Labor Law & Policy J*. 1, at 6–7.

6 International Labour Organization, Convention Concerning Forced or Compulsory Labour (No. 29), 39 UNTS 55, adopted 28 June 1930, entered into force 1 May 1932, Art. 2, at <https://www.ilo.org/dyn/normlex/en/f?p=NORMLEXPUB:12100:0::NO::P12100_ILO_CODE:C029>.

7 C. Costello, ‘Migrants and Forced Labour: A Labour Law Response’ in *The Autonomy of Labour Law*, eds A. Bogg et al. (2015) 189, at 202, 207, 216.

8
*Chowdury and Others* v. *Greece* (2017) Application No. 21884/15 ECtHR; *R* v. *Khan* [2010] EWCA Crim 2880.

9 UN Committee on Economic, Social and Cultural Rights, *General Comment No. 18: The Right to Work (Art. 6 of the Covenant)* (2006) UN Doc E/C.12/GC/18, paras 3, 4, 6, 9, at <https://www.refworld.org/docid/4415453b4.html>.

10 P. Gilabert, ‘Dignity at Work’ in *Philosophical Foundations of Labour Law*, eds H. Collins et al. (2018) 68, at 80; D. Ashiagbor, ‘Article 15: Freedom to Choose an Occupation and Right to Engage in Work’ in *The EU Charter of Fundamental Rights: A Commentary*, eds S. Peers et al. (2014) 423, at 425.

11 K. Calavita, *Inside the State: The Bracero Program, Immigration, and the INS* (1992) 58.

12 M. Ruhs, *The Price of Rights: Regulating International Labor Migration* (2013); H. Shamir, ‘The Paradox of “Legality”: Temporary Migrant Worker Programs and Vulnerability to Trafficking’ in *Revisiting the Law and Governance of Trafficking, Forced Labor and Modern Slavery*, ed. P. Kotiswaran (2017) 471, at 481–485.

13 On the continuum of exploitation approach, see K. Skrivankova, *Between Decent Work and Forced Labour: Examining the Continuum of Exploitation* (2010), at <https://www.jrf.org.uk/report/between‐decent‐work‐and‐forced‐labour‐examining‐continuum‐exploitation>; J. Fudge, ‘Modern Slavery, Unfree Labour and the Labour Market: The Social Dynamics of Legal Characterization’ (2018) 27 *Social & Legal Studies* 414, at 420; Shamir, id.

14 A. Paz‐Fuchs, ‘Badges of Modern Slavery’ (2016) 79 *Modern Law Rev*. 757, at 775; V. Stoyanova, *Human Trafficking and Slavery Reconsidered: Conceptual Limits and States’ Positive Obligations in European Law* (2017) 278. See also V. E. Munro, ‘Of Rights and Rhetoric: Discourses of Degradation and Exploitation in the Context of Sex Trafficking’ (2008) 35 *J. of Law and Society* 240, at 258–259.

15 J. O'Connell Davidson, ‘Troubling Freedom: Migration, Debt, and Modern Slavery’ (2013) 1 *Migration Studies* 176, at 183.

16 United Nations Office on Drugs and Crime, *The Role of Recruitment Fees and Abusive and Fraudulent Recruitment Practices of Recruitment Agencies in Trafficking in Persons* (2015) 9, at <https://www.unodc.org/documents/human‐trafficking/2015/Recruitment_Fees_Report‐Final‐22_June_2015_AG_Final.pdf>.

17 Calavita, op. cit., n. 11, p. 74; A. Kemp and R. Raijman, *Migrants and Workers: The Political Economy of Labor Migration in Israel* (2008) 101.

18 Calavita, id., pp. 74–75; Kemp and Raijman, id., pp. 100–101; Albin, op. cit., n. 3, p. 135.

19 M. R. Freedland and C. Costello, ‘Migrants at Work and the Division of Labour Law’ in *Migrants at Work: Immigration and Vulnerability in Labour Law*, eds C. Costello and M. R. Freedland (2014) 1, at 8; G. Mundlak, ‘Neither Insiders Nor Outsiders: The Contractual Construction of Migrant Workers’ Rights and the Democratic Deficit’ (2003) 27 *Tel Aviv University Law Rev*. 423.

20 Shamir, op. cit., n. 12, p. 480.

21 Id., p. 485.

22 Albin, op. cit., n. 3, p. 138.

23 Id., p. 135. See also Fudge and Tham, op. cit., n. 5, pp. 5–6.

24 B. Anderson and M. Ruhs, ‘Migrant Workers: Who Needs Them? A Framework for the Analysis of Staff Shortages, Immigration, and Public Policy’ in *Who Needs Migrant Workers? Labour Shortages, Immigration, and Public Policy*, eds M. Ruhs and B. Anderson (2010) 15, at 31.

25 Calavita, op. cit., n. 11, p. 55.

26 International Covenant on Civil and Political Rights, 999 UNTS 171, adopted 16 December 1966, entered into force 23 March 1976, Art. 8(3)(c), at <https://www.ohchr.org/en/instruments‐mechanisms/instruments/international‐covenant‐civil‐and‐political‐rights>.

27 Historically, the definition of forced labour and the exceptions to it were meant to enable colonial governments to benefit from the labour of ‘natives’ in the colonies. See Stoyanova, op. cit., n. 14, pp. 195–198.

28
*Van der Mussele* v. *Belgium* (1983) Application No. 8919/80 ECtHR 13, para. [37].

29 International Labour Organization, Migrant Workers (Supplementary Provisions) Convention (No. 143) 1975, Art. 14, at <https://www.ilo.org/dyn/normlex/en/f?p=NORMLEXPUB:12100:0::NO::P12100_ILO_CODE:C143>. See also J. Fudge. ‘Precarious Migrant Status and Precarious Employment: The Paradox of International Rights for Migrant Workers’ (2012) 34 *Comparative Labor Law and Policy J*. 95, at 124.

30 Costello, op. cit., n. 7, p. 210.

31 On the position of migrants under international human rights treaties, see for example UN Human Rights Committee, *CCPR General Comment No. 15: The Position of Aliens under the Covenant* (1986) paras 4, 7, at <https://www.refworld.org/docid/45139acfc.html>; UN Committee on Economic, Social and Cultural Rights, op. cit., n. 9, paras 18, 23.

32 Ruhs, op. cit., n. 12; M. Ruhs and P. Martin, ‘Numbers vs. Rights: Trade‐Offs and Guest Worker Programs’ (2008) 42 *International Migration Rev*. 249.

33 Ruhs, id., p. 165.

34 State Comptroller and Ombudsman of Israel, *State Comptroller Annual Report 65A: Ministry of Interior, Administration of Border Crossings, Population and Immigration, Employment of Palestinian Workers in the Construction Industry in Israel* (2014) 491, at <https://www.mevaker.gov.il/he/Reports/Report_266/fb860755‐1b0b‐4206‐b1ab‐c76ccf254fe0/210‐ver‐4.pdf>.

35 Kemp and Raijman, op. cit., n. 17, pp. 53–55, p. 80.

36 Population and Immigration Authority, *Foreigners in Israel Report* (2020) 25, at <https://www.gov.il/BlobFolder/generalpage/foreign_workers_stats/he/%D7%A0%D7%AA%D7%95%D7%A0%D7%99%20%D7%96%D7%A8%D7%99%D7%9D%20%D7%91%D7%99%D7%A9%D7%A8%D7%90%D7%9C%20%D7%A8%D7%91%D7%A2%D7%95%D7%9F%201%202020.pdf>; G. Nathan, *Annual Report International Migration: Israel 2019–2020* (2020) 69, 72, 74. According to the Population and Immigration Authority report, there are about 30,000 asylum seekers (not all of them working) and 48,000 tourists who have overstayed their visa in Israel.

37 Entry into Israel Regulations 5734‐1974; HCJ 4542/02 *Kav LaOved (‘The Workers’ Hotline’)* v. *Government of Israel* (30 March 2006) (‘the *Binding Arrangement* case’) para. 15.

38 Kemp and Raijman, op. cit., n. 17, p. 101; Mundlak, op. cit., n. 19, p. 443.

39 Mundlak, id., p. 470.

40 The *Binding Arrangement* case, op. cit., n. 37, para. 27.1 of the Petition. The term ‘runaway’ was used by some employers as well as some public officials in reference to workers who left their registered employer. Despite strong criticism by the National Labour Court, the term is still occasionally used.

41 Prime Minister's Decision of 3 October 2002; Regulation of the Immigration Authority, 7 January 2003 – ‘Closed Sky Regulation.’

42 S. Willen, ‘Citizens, “Real” Others, and “Other” Others: The Biopolitics of Otherness and the Deportation of Unauthorized Migrant Workers from Tel Aviv, Israel’ in *The Deportation Regime: Sovereignty, Space, and the Freedom of Movement*, ed. N. De Genova (2010) 273, at 281–282; A. Gill and Y. Dahan, ‘Between Neo‐Liberalism and Ethno‐Nationalism: Theory, Policy and Law in the Deportation of Migrant Workers in Israel’ (2006) 10 *Law and Government* 347, at 352, 366–367, 375.

43 ‘Closed Sky Regulation’, op. cit., n. 41.

44 Compare to the *Rantsev* case, op. cit., n. 4, paras 20, 298.

45 The Knesset's Migrant Workers Committee, 9 November 2004, Maasiyahu Prison Commander Rami Ovadia.

46 Population Administration, ‘Credit Procedure for Workers Abandoning Their Employers’, 9 October 2002.

47 Mundlak, op. cit., n. 19, p. 447.

48 The *Binding Arrangement* case, op. cit., n. 37.

49 Id., paras 46–55. On sectoral regulation and labour migration, see also Albin, op. cit., n. 3.

50 The *Binding Arrangement* case, op. cit., n. 37, paras 12, 21. The Association of Contractors and Builders and the Association of Flower Growers were two of the original respondents, alongside the Government of Israel, the Minister of Interior, and the Minister of Labour and Welfare.

51 Id., para. 2 of the Government's Response of 20 January 2005. See also Government Decision 2446, ‘Regulating the Employment of Foreign Workers’, 15 August 2004, at <https://www.gov.il/he/departments/policies/2004_des2446>.

52 The *Binding Arrangement* case, op. cit., n. 37, paras 29, 39 of Judge Levy's opinion.

53 Id., para. 30.

54 Id., para. 35.

55 Id., para. 28.

56 Id., para. 29.

57 Id., para. 32.

58 Id., para. 31.

59 Id., para. 32.

60 Id., para. 4 of Vice‐President Emeritus Cheshin's judgment.

61 M. Niezna, ‘Binding’ in *An Alternative Anti‐Trafficking Action Plan: A Proposed Model Based on a Labor Approach to Trafficking*, eds H. Shamir and M. Niezna (2020) 38, at 39–41.

62 Government Decision 447 ,‘Change in the Employment Model of Foreign Workers in the Agriculture Sector’, 12 September 2006, at <https://www.gov.il/he/departments/policies/2006_des447>.

63 Id., paras 4(a), 4(q).

64 Y. Kurlander, ‘The Marketization of Migration: On the Emergence, Flourishment and Change of the Recruitment Industry for Agricultural Migrant Workers from Thailand to Israel’ (2019) PhD thesis, Haifa University, p. 144.

65 Id. See also Y. Kurlander, ‘Placement and Manpower Agencies’ in eds Shamir and M. Niezna, op. cit., n. 61, p. 64.

66 Interview with Adv. Michal Tadjer, lawyer at Kav LaOved (31 December 2018); Kurlander, op. cit., n. 64, p. 185.

67 The *Binding Arrangement* case, op. cit., n. 37, Decisions of 17 November 2008 and 24 September 2009.

68 Id., Decision of 3 December 2009, para. 1.

69 T. Kritzman‐Amir, ‘Narratives and Social Change in the Opinions of the Supreme Court on the Matter of Visas as Applied to Migrant Workers in Israel: From the Binding Case to the Pregnant Migrant Worker Case’ (2014) 18 *Law and Business* 509, at 523–530.

70 HCJ 10843/04 *Hotline for Migrant Workers* v. *Government of Israel* (19 September 2007) (‘the *Yilmazlar* case’).

71 Id., para. 1. Employment with a company based in the workers’ home state and carrying out work in the host state is not unique to Israel. The regime of ‘posted workers’ in the European Union is a notable example, though in a very different political and legal context, where concerns of ‘social dumping’, the impact on local workers, and considerations of free trade are more significant than in the Israeli case, and the tying to an individual employer less so. A comprehensive review of the literature concerning posted workers regimes is beyond the scope of this article. See for example J. Fudge and G. Mundlak, ‘Justice in a Globalizing World: Resolving Conflicts Involving Workers Rights beyond the Nation State’ (2013) EUI Working Papers 2013/06, at <https://cadmus.eui.eu/handle/1814/27014>; J. Arnholtz and N. Lillie (eds), *Posted Work in the European Union: The Political Economy of Free Movement* (2020).

72 The *Yilmazlar* case, para. 11. See also State Response of 21 January 2005.

73 The *Binding Arrangement* case, op. cit., n. 37, para. 16 of the Supplementary Statement of the Respondents of 21 May 2003; para. 27 of the Response to the Petition of 31 December 2003.

74 HCJ 1834/09 *Doron* v. *Minister of Interior* (16 October 2013) ‘the *Doron* case’) Protocol of 1 March 2010.

75 H. Ben‐Israel, ‘“The Fragile String to Life Itself”: Labor, Migration and Care between Altruism and Instrumentalism’ (2018) LL.M thesis, Tel‐Aviv University, pp. 53–55.

76 Entry into Israel Regulations (Determination of Geographical Areas for the Employment of Foreign Caregivers) 5774‐2014, at <https://www.nevo.co.il/law_html/law00/132991.htm>.

77 Entry into Israel Regulations (Supervision of the Changes of Employment of Foreign Caregivers) 5774‐2014, at <https://www.nevo.co.il/law_html/law00/132992.htm>.

78 Entry into Israel Law 5712‐1952, art. 3A(b), at <https://www.nevo.co.il/law_html/law01/189_003.htm#Seif45>.

79 Interview with Tadjer, op. cit., n. 66.

80 Ben‐Israel, op. cit., n. 75, p. 48.

81 Compare *Van der Mussele*, op. cit., n. 28, para. 37; Paz‐Fuchs, op. cit., n. 14, p. 775.

82 The *Yilmazlar* case, op. cit., n. 70, para. 13 of Vice‐President Rivlin's judgment.

83 Id., para. 3; para. 19 of Judge Levy's judgment.

84 Id., para. 14.

85 Id., para. 19 of Judge Levy's judgment; paras 76, 80–86 of the Petition.

86
*Adem Tavsan*, op. cit., n. 1.

87 The *Doron* case, op. cit., n. 74, p. 3 of Protocol of 1 March 2010.

88 Kritzman‐Amir, op. cit., n. 69, p. 529.

89 The *Yilmazlar* case, op. cit., n. 70, para. 30.

90 HCJ 2385/16 *National Labor Federation* v. *Government of Israel* (3 August 2016).

91 The *Yilmazlar case*, op. cit., n. 70, para. 18 of Vice‐President Rivlin's judgment.

92 The *Doron* case, op. cit., n. 74, paras 7–9 of the Petition.

93 Interview with Adv. Oded Feller, Association for Civil Rights in Israel (6 January 2020).

94 Kritzman‐Amir, op. cit., n. 69, p. 537.

95 Interview with Tadjer, op. cit., n. 66.

96 See for example the *Binding Arrangement* case, op. cit., n. 37, paras 30–38 of the State Response of 31 December 2003.

97 Compare to the definition of ‘frontier worker’ under the International Convention on the Protection of the Rights of All Migrant Workers and Members of Their Families. United Nations Office of the High Commissioner for Human Rights, International Convention on the Protection of the Rights of All Migrant Workers and Members of Their Families, 2220 UNTS 3, adopted 18 December 1990, entered into force 1 July 2003, Art. 2(a), at <https://www.ohchr.org/en/instruments‐mechanisms/instruments/international‐convention‐protection‐rights‐all‐migrant‐workers>.

98 L. Farsakh, *Labour Migration and the Palestinian State: The Political Economy of Palestinian Labour Flows to Israel* (2005) 30, 37; A. Paz‐Fuchs and Y. Ronen, ‘Occupational Hazards’ (2012) 30 *Berkeley J. of International Law* 580, at 629–630.

99 Paz‐Fuchs and Ronen, id., pp. 629–632.

100 Farsakh, op. cit., n. 98, p. 105; COGAT Order, *Status of Permits for Palestinians to Enter Israel, Depart Abroad, and Move between the Judea and Samaria Region and the Gaza Strip* (2019) 3, 6, at <https://www.gisha.org/UserFiles/File/LegalDocuments/procedures/general/50.pdf>.

101 See Population and Immigration Authority, *PIBA Temporary Regulation for the Management of Employment of Palestinian Workers in the Industry and Services Sector*, 9.1.3001, updated 25 February 2018, paras B.3, C.3, F.2, F.5, at <https://www.industry.org.il/files/work/law/%d7%a0%d7%95%d7%94%d7%9c%20%d7%96%d7%9e%d7%a0%d7%99%2025_2_18.pdf>; Ministry of Construction and Housing, *Allocation of Employment Permits to Palestinian Construction Workers: Conclusions of the Inter‐Ministerial Team Work* (2016) 13.

102 Population and Immigration Authority, id., paras H.12, H.13.

103 Y. Berda, *The Bureaucracy of the Occupation: The Permit Regime in the West Bank, 2000–2006* (2012) 97, 101.

104 J. Preminger, *Labor in Israel: Beyond Nationalism and Neoliberalism* (2018) 167.

105 Ministry of Construction and Housing, op. cit., n. 101, p. 14.

106 Id.

107 Id.; interview with DD, trade union representative (10 February 2019).

108 M. Niezna and Kav LaOved, *The Occupation of Labor: Palestinian Employment in Israel* (2018) 19, at <https://www.kavlaoved.org.il/en/the‐occupation‐of‐labor‐palestinian‐employment‐in‐israel/>.

109 Id.; H. Etkes and W. Adnan, *Illegal Trafficking in Permits for Palestinian Workers in Israel: The Status Quo and Planned Reform* (2019), at <https://www.boi.org.il/en/NewsAndPublications/PressReleases/Pages/25‐9‐2019.aspx>.

110 HCJ 6272/20 *Kav LaOved* v. *Government of Israel* (‘the *Palestinian binding* case’) Petition from 10 September 2020.

111 Government Decision 2174, ‘Increasing the Scope of Employment in Israel of Palestinian Workers from the Judea and Samaria Region, Streamlining the Allocation of Work Permits, and Guaranteeing Fair Working Conditions for Palestinian Workers’, 18 December 2016, para. 3(e), at <https://www.gov.il/he/Departments/policies/2016_dec2174>.

112 Ministry of Construction and Housing, op. cit., n. 101, pp. 24–25.

113 Population and Immigration Authority, ‘Regulation of Employment of Palestinian Workers in the Construction Sector’, 22 October 2020.

114 Niezna and Kav LaOved, op. cit., n. 108. In 2016, about 12,500 Palestinian workers held such permits.

115 M. Niezna, ‘Under Control: Palestinian Workers in Israel during COVID‐19’ *Border Criminologies*, 7 July 2020, at <https://www.law.ox.ac.uk/research‐subject‐groups/centre‐criminology/centreborder‐criminologies/blog/2020/07/under‐control>.

116 International Labour Organization, *ILO Indicators of Forced Labour* (2012) 17, at <https://www.ilo.org/wcmsp5/groups/public/—asia/—ro‐bangkok/documents/publication/wcms_345673.pdf>.

117 HCJ 2730/20 *Kav LaOved* v. *Minister of Health* (24 September 2020), Preliminary Response of 5 May 2020, para. 7.

118 Interview with GG, former Kav LaOved representative (20 December 2019).

119 Kav LaOved, *Migrant and Palestinian Workers in Israel: Shadow Report Submitted to the Human Rights Council's Universal Periodic Review (Israel Session)* (2018), at <https://lib.ohchr.org/HRBodies/UPR/Documents/Session3/IL/KLO_ISR_UPR_S3_2008_KavLaOved_uprsubmission.pdf>.

120 Interview with FF, former NGO representative (21 July 2019); interview with DD, op. cit., n. 107.

121 Interview with FF, id.

122
*Kav LaOved* v. *Government of Israel*, op. cit., n. 110, affidavit of Adv. Khaled Duhi, para. [6].

123 Interview with FF, op. cit., n. 120.

124 Id.; interview with DD, op. cit., n. 107.

125 Interview with DD, id.

